# Whole-genome sequencing and comparative analysis of two plant-associated strains of *Rhodopseudomonas palustris* (PS3 and YSC3)

**DOI:** 10.1038/s41598-018-31128-8

**Published:** 2018-08-24

**Authors:** Kai-Jiun Lo, Shih-Shun Lin, Chia-Wei Lu, Chih-Horng Kuo, Chi-Te Liu

**Affiliations:** 1 Institute of Biotechnology, National Taiwan University, Taipei, 106 Taiwan; 2 Agricultural Biotechnology Research Center, Academia Sinica, Taipei, 115 Taiwan; 3 Institute of Plant and Microbial Biology, Academia Sinica, Taipei, 115 Taiwan; 4 Molecular and Biological Agricultural Sciences Program, Taiwan International Graduate Program, National Chung Hsing University and Academia Sinica, Taipei, 115 Taiwan; 5 Graduate Institute of Biotechnology, National Chung Hsing University, Taichung City, 402 Taiwan; 6 Center of Biotechnology, National Taiwan University, Taipei, 106 Taiwan; 7 National Center for High-Performance Computing, National Applied Research Laboratories, Hsinchu, 300 Taiwan; 8 Center for Shrimp Disease Control and Genetic Improvement, National Cheng Kung University, Tainan, 701 Taiwan

## Abstract

*Rhodopseudomonas palustris* strains PS3 and YSC3 are purple non-sulfur phototrophic bacteria isolated from Taiwanese paddy soils. PS3 has beneficial effects on plant growth and enhances the uptake efficiency of applied fertilizer nutrients. In contrast, YSC3 has no significant effect on plant growth. The genomic structures of PS3 and YSC3 are similar; each contains one circular chromosome that is 5,269,926 or 5,371,816 bp in size, with 4,799 or 4,907 protein-coding genes, respectively. In this study, a large class of genes involved in chemotaxis and motility was identified in both strains, and genes associated with plant growth promotion, such as nitrogen fixation-, IAA synthesis- and ACC deamination-associated genes, were also identified. We noticed that the growth rate, the amount of biofilm formation, and the relative expression levels of several chemotaxis-associated genes were significantly higher for PS3 than for YSC3 upon treatment with root exudates. These results indicate that PS3 responds better to the presence of plant hosts, which may contribute to the successful interactions of PS3 with plant hosts. Moreover, these findings indicate that the existence of gene clusters associated with plant growth promotion is required but not sufficient for a bacterium to exhibit phenotypes associated with plant growth promotion.

## Introduction

In 1978, Kloepper and Schroth proposed the concept of plant growth-promoting rhizobacteria (PGPRs)^1^. PGPRs are a diverse subgroup of rhizosphere-colonizing bacteria that can have beneficial effects on soil quality and crop growth and can sustain soil health via various mechanisms^1^. PGPRs can facilitate plant growth by increasing nutrient availability (e.g., nitrogen fixation) and nutrient solubilization (e.g., phosphate solubilization) and by producing phytohormones such as indole acetic acid (IAA), 2,3-butanediol, and cytokinins^2–4^. In addition, PGPRs can improve plant tolerance to environmental stress by metabolizing 1-aminocyclopropane-1-carboxylic acid (ACC), a precursor of ethylene, as a stress hormone^2–4^. Moreover, PGPR can also protect plants from pathogen infection by producing antibiotics or activating induced systemic resistance^5^. Due to these properties of PGPRs, these bacteria are widely used as biofertilizers and biocontrol agents^3^.

*Rhodopseudomonas palustris* is a phototrophic purple non-sulfur bacterium (PNSB) that can sustain itself in different metabolic states, including photoautotrophic, photoheterotrophic, chemoautotrophic and chemoheterotrophic states^6^. This bacterium has been widely used in industrial applications for bioremediation and sewage treatment and for the removal of phytotoxic compounds^7,8^. In addition, this bacterium can convert complex organic compounds into biomass and bioenergy using substrates that are plant-derived compounds, pollutants, or aromatic compounds^6,9–12^. Some studies have indicated that *R. palustris* can also be used as a biofertilizer to improve crop yield^13–15^. *R. palustris* strain PS3 can have beneficial effects on plant growth and can enhance the efficiency of fertilizers used in either soil or hydroponic cultivation systems^13,16^. Although PS3 is a promising PGPR, genomic information and the underlying molecular mechanisms for plant growth promotion (PGP) by PS3 have yet to be ascertained.

Systematic analysis of whole-genome sequences is a powerful approach to identify either causal genes that contribute to plant growth-promoting activities or potential PGPR candidates^17–21^. Some studies have conducted genomic analyses of *R. palustris* strains such as CGA009, HaA2, BisB18, and TIE^6,22,23^. However, none of these strains are plant-associated strains. In this study, we performed a genomic characterization of two plant-associated *R. palustris* strains. One strain is the effective PGPR strain PS3, and the other is YSC3, which has been shown to be ineffective in PGP^13^. To elucidate the potential modes of action via which *R. palustris* PS3 has beneficial effects on plants, we compared the genomic compositions of these two strains as well as that of *R. palustris* CGA009, which is the genomic representative of this species, and the sequence derived from the NCBI database. We focused on genes involved in carbohydrate and nitrogen metabolism, phosphate solubilization, phytohormone production, biofilm formation, chemotaxis, and plant colonization.

## Results

### General characteristics of the genomes

**Figure 1:**
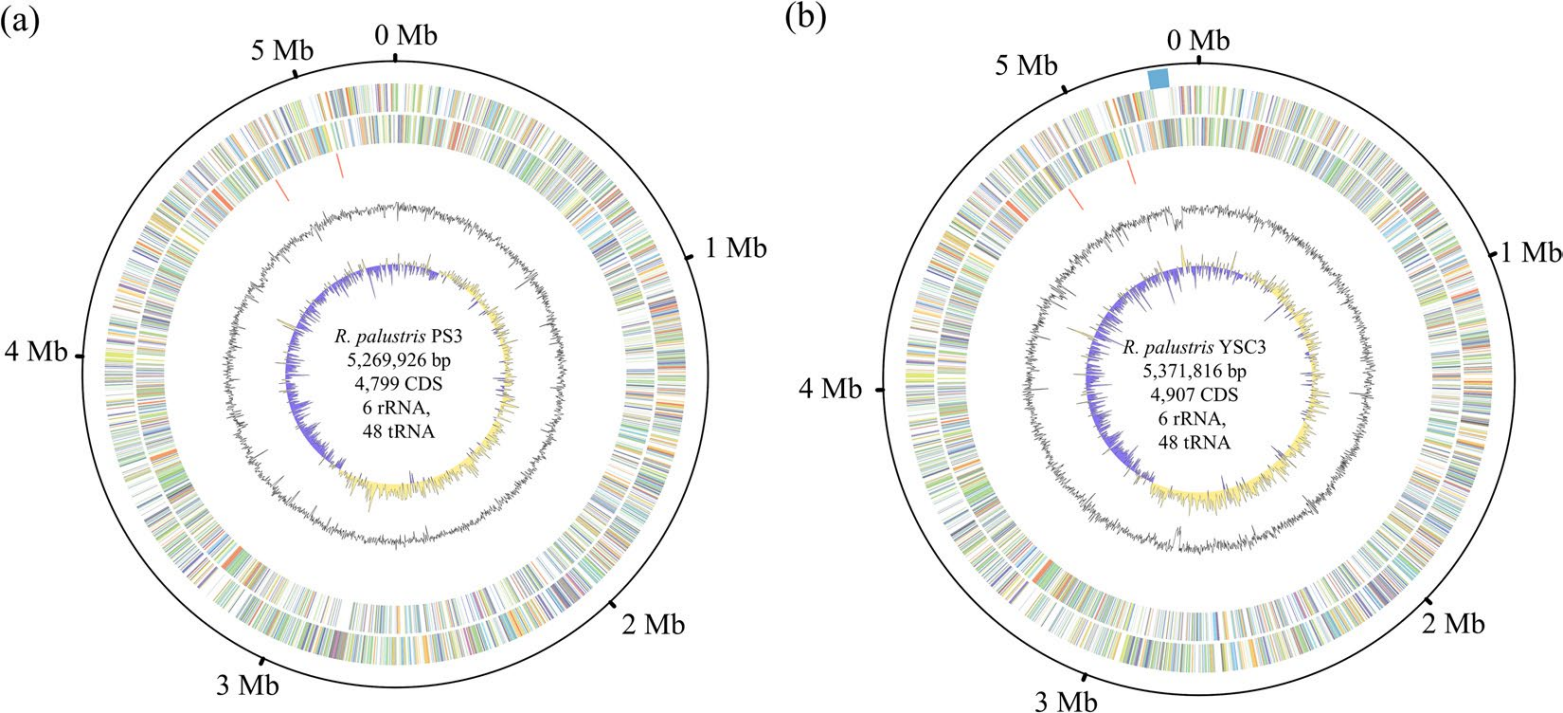
Genome map of *Rhodopseudomonas palustris* PS3 (**a**) and YSC3 (**b**). Rings from the outside as follows: (1) scale marks (unit, Mb), (2) genomic island, (3) protein-coding genes on the forward strand colored by COG category, (4) protein-coding genes on the reverse strand (same color scheme as the second circle), (5) rRNA genes, (6) GC content (deviation from average), and (7) GC skew in blue (below average) and yellow (above average).

The general genomic features of the *R. palustris* strains are summarized in Table 1 and Fig. 1. All three strains have a single circular chromosome that is ~5.3 Mb and encodes 6 rRNAs and ~4,800–4,900 protein-coding genes. CGA009 has an additional tRNA gene annotated as tRNA-OTHER, which may be an artifact. CGA009 harbors one 8.4-kb circular plasmid^6^, while PS3 and YSC3 do not harbor any plasmid. Based on the anomalous G + C contents determined by an online tool, Zisland Explorer^24^, we found that no horizontally transferred genomic island of DNA is present in the PS3 and CGA009 genomes. Furthermore, Larimer *et al*. also indicated that there were no horizontally transferred genomic islands in the genome of *R. palustris* CGA009^6^. In contrast, YSC3 contained one 56-kb genomic island located at 5,229,652–5,285,897 bp (Fig. 1). This genomic island contains 60 protein-coding genes (locus tags: RPYSC3_47720-48310), and all of these genes encode hypothetical proteins.

**Table 1:** General features of sequenced strains of *Rhodopseudomonas palustris*.

Feature	PS3	YSC3	CGA009
Accession	CP019966	CP019967	NC_005296.1
Size (bp)	5,269,926	5,371,816	5,459,213
G + C content	65.3%	65.2%	65.0%
rRNA	6	6	6
tRNA	48	48	49
Protein-coding genes	4,799	4,907	4,841
Hypothetical genes	942	1,331	1,379
Plasmid	none	none	1
Reference	This study	This study	^6^

The characteristics of the genomes of *R. palustris* PS3 and YSC3 were analyzed in this study, and genomic data pertaining to *R. palustris* CGA009 (GenBank accession no. NC_005296) were downloaded from the NCBI database.

**Figure 2:**
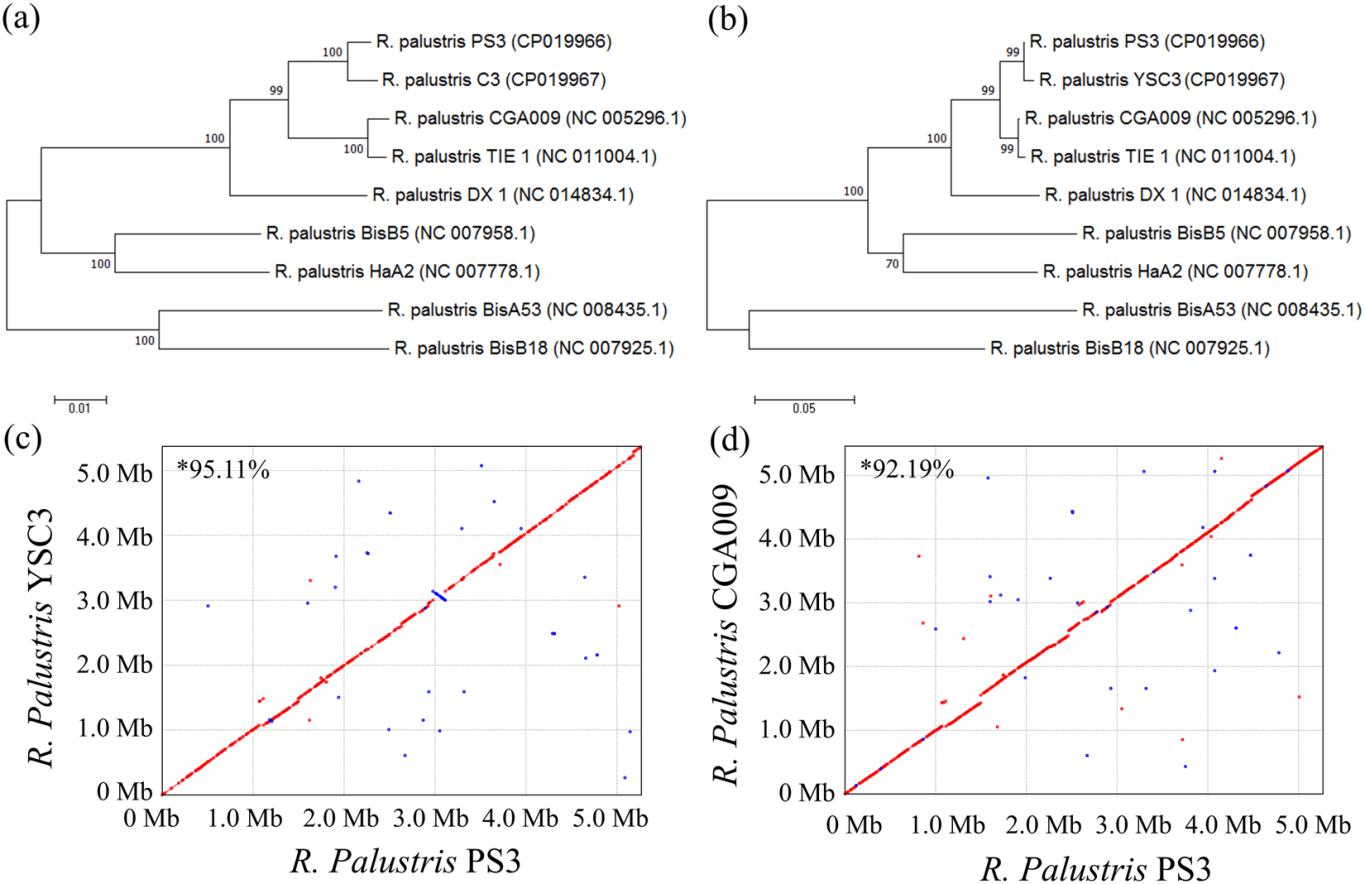
Phylogenetic tree and genome alignments of *R. palustris* based on housekeeping and functional genes and nucleotide levels. Maximum-likelihood tree based on concatenated *recA-rpoB-dnaK* gene sequences (**a**) and *puf* gene sequences (**b**) showing the relationships among the *R. palustris* strains. Both bootstrap values (1000 replicates) are given at branch points and generated in MEGA7. (**c**,**d**) Pairwise genome alignments among *R. palustris* PS3, YSC3 and CGA009. Synteny plots show the comparison of the PS3 genome (c and d, vertical axis) with the genomes of YSC3 (c, horizontal axis) and CGA009 (d, horizontal axis). Forward matches are plotted in red, and reverse matches are plotted in blue. The nucleotide sequence similarities were calculated by software MUMmer^100^ with the nucmer function and represented as “*”.

The phylogenetic tree of the nine *R. palustris* strains was constructed based on concatenated multilocus sequence analysis (MLSA) of three housekeeping genes (i.e., *recA*, *rpoB*, and *dnaK*; the concatenated alignment is ~7 kb) and is shown in Fig. 2a. The strains were divided into five major groups. All of the *R. palustris* reference strains shared >91% nucleotide sequence identity with PS3; YSC3 was the most closely related strain, with a >99% identity, while CGA009 and TIE-1 exhibited >97% identity. We also constructed a phylogenetic tree based on the *pufL* and *pufM* genes (Fig. 2b). These two genes encode the two subunits of the light reaction center core protein and have been used as markers for phylogenetic analysis within the genus *Rhodopseudomonas*^13,25^. As shown in Fig. 2b, the tree topology is identical to that of the tree generated based on the three housekeeping genes, which is consistent with PS3 and YSC3 being closely related (bootstrap support >99%). At the genomic level, we identified a total of 2,515 single-copy genes that were conserved among all the *R. palustris* strains compared. Based on these genes, we calculated the average nt/aa similarity among the strains, and four main clusters were formed in the phylogenetic tree of *R. palustris* (Supplementary Fig. S1). PS3 had a close phylogenetic relationship with YSC3, while the CGA009 and TIE strains were grouped in a nearby cluster. Based on these results, we included CGA009 as a reference strain in our comparative genomic analysis. CGA009, the first sequenced *R. palustris* strain, is a representative model strain, and the sequence of this strain is available in the NCBI database.

**Figure 3:**
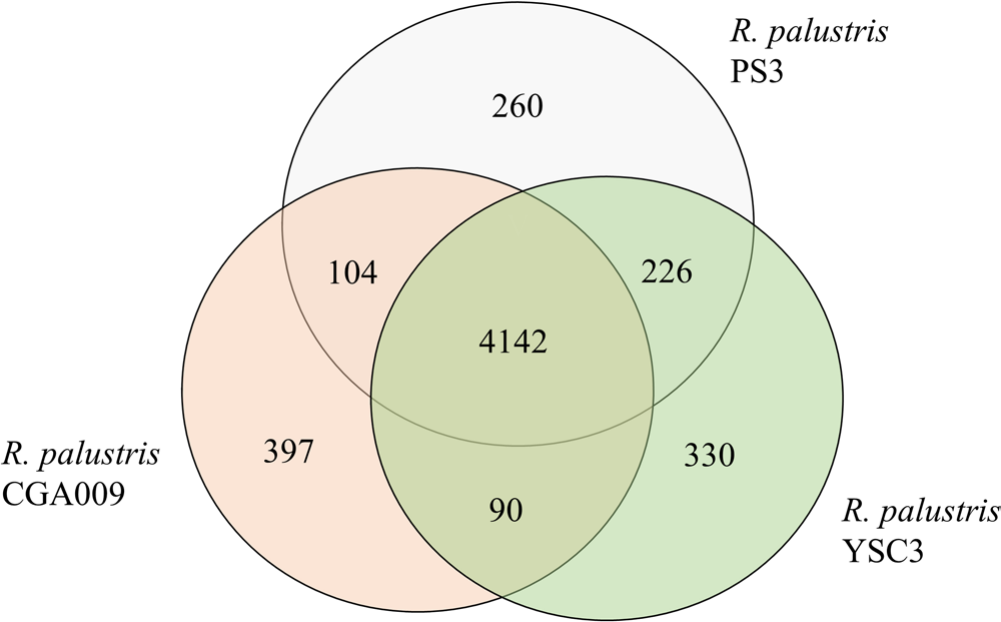
Distribution patterns of homologous gene clusters. The homologous gene clusters are those located at the intersection of *R. palustris* PS3, YSC3 and CGA009.

**Figure 4:**
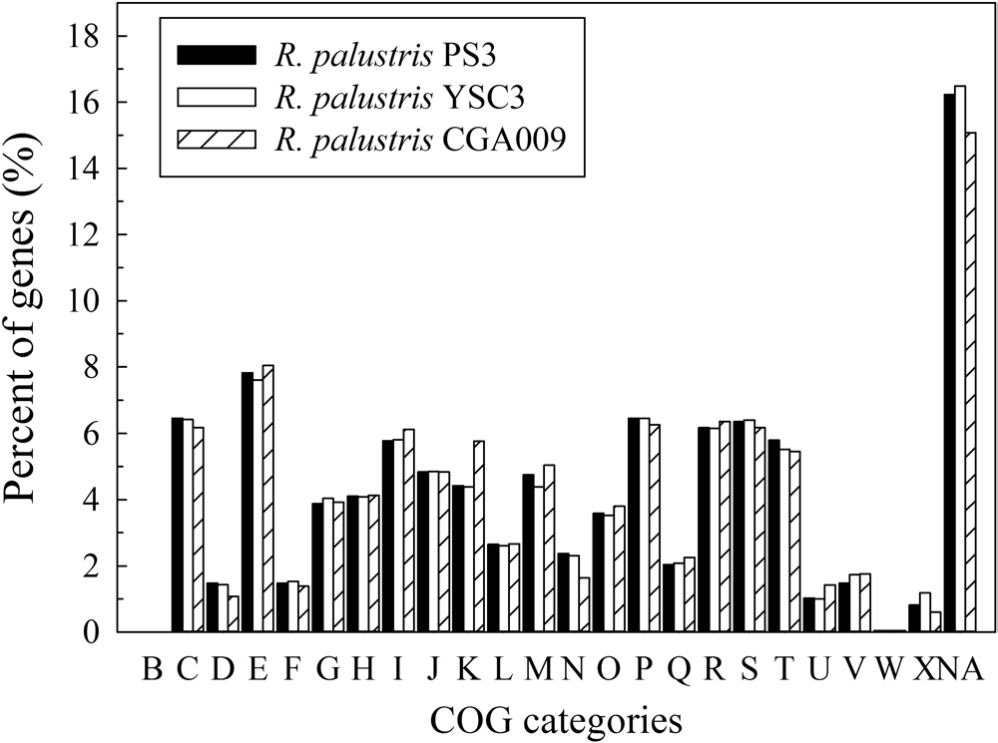
COG categories in the three *R. palustris* strains. Functional categorization of genes was performed by the COG database^92^. The y-axis indicates the percentage of genes assigned with the COG category relative to all genes. The x-axis represents the COG functional category. The groups in each COG category are as follows: (B) chromatin structure and dynamics; (C) energy production and conversion; (D) cell cycle control, cell division and chromosome partitioning; (E) amino acid transport and metabolism; (F) nucleotide transport and metabolism; (G) carbohydrate transport and metabolism; (H) coenzyme transport and metabolism; (I) lipid transport and metabolism; (J) translation, ribosomal structure and biogenesis; (K) transcription; (L) replication, recombination and repair; (M) cell wall/membrane/envelope biogenesis; (N) cell motility; (O) posttranslational modification, protein turnover and chaperones; (P) inorganic ion transport and metabolism; (Q) secondary metabolite biosynthesis, transport and catabolism; (R) general function prediction only; (S) function unknown; (T) signal transduction mechanisms; (U) intracellular trafficking, secretion and vesicular transport; (V) defense mechanisms; (W) extracellular structures; (X) mobilome: prophages and transposons; and (NA) no COG assignment.

Pairwise genome alignments demonstrated that PS3 shares 95.11% identity with YSC3 at the nucleotide level and exhibits a high level of synteny conservation (Fig. 2c). In contrast, CGA009 shares 92.19% identity with PS3 (Fig. 2d). This result is consistent with the phylogenetic tree analysis (Fig. 2a,b and Supporting Information Fig. S1). Gene homology analysis showed that the genomes of PS3, YSC3 and CGA009 were composed of 5,549 orthologous gene clusters, and 4,142 clusters were conserved among the 3 strains (Fig. 3 and Supplementary Table S1). On the other hand, 226 gene clusters were shared by PS3 and YSC3, while only 104 gene clusters were shared by PS3 and CGA009 (Fig. 3). There were 260 gene clusters that were unique to the PS3 strain (Supplementary Table S2). Sixty percent of these genes encode hypothetical proteins, and the remaining 40% include genes such as those encoding the urea ABC transporters (*UrtACD*, RPPS3_10430, RPPS3_10440 and RPPS3_10450). The list of unique gene clusters in strains YSC3 and CGA009 are shown in Supplementary Tables S3 and S4, respectively. As shown in Fig. 4, the distribution patterns of the Clusters of Orthologous Groups (COG)-assigned proteins for these three strains highly resemble each other. Detailed analyses for the annotation of protein-coding genes in PS3 and YSC3 are shown in Supplementary Tables S5 and S6, respectively. Notably, only CGA009 has a gene belonging to the COG B group (i.e., chromatin structure and dynamics), which is associated with acetylpolyamine aminohydrolase^6^. It has been suggested that acetoin can be converted to acetate by acetylpolyamine aminohydrolase upon carbon source depletion^26^. We noticed that CGA009 exhibited a higher percentage of genes in the COG K group (i.e., transcription) than did PS3 or YSC3. We further analyzed the gene contents in the K group and found that CGA009 contains a larger ratio of genes encoding transcriptional regulators (Supplementary Table S7).

### Carbon source utilization

**Figure 5:**
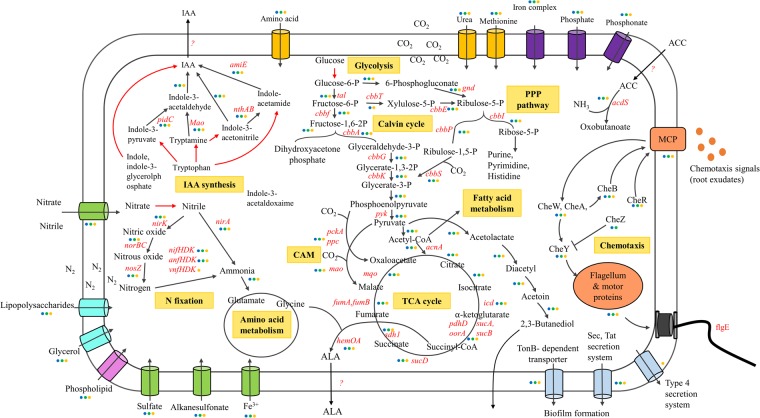
Schematic depiction of genes involved in metabolism (PPP, TCA cycle, glycolysis, nitrogen assimilation), rhizosphere adaptation and plant growth promotion in *R. palustris*. Genes annotated in each strain are marked with colored circles representing PS3 (blue), YSC3 (green), and CGA009 (orange). Red arrows represent nonspecific genes that were annotated among the three strains. “?” represents unknown function.

*R. palustris* can obtain carbon from carbon dioxide and/or organic compounds^6^. Core metabolic pathways, such as the Calvin-Benson-Bassham (CBB) pathway of CO_2_ fixation, the complete tricarboxylic acid (TCA) cycle, an Embden-Meyerhof-Parnas (EMP) pathway and a pentose phosphate pathway (PPP), were found in the genomes of both PS3 and YSC3 (Fig. 5). Larimer *et al*. reported that *R. palustris* CGA009 lacks genes encoding hexokinase^6^, which is also the case for PS3 and YSC3. CGA009 can catabolize a variety of aromatic compounds for growth^27^, and we obtained similar results for the *R. palustris* derivatives^22^. Several genes in the PS3 and YSC3 strains encode dioxygenases involved in the degradation of aromatic compounds, such as 3,4-dihydroxyphenylacetate 2,3-dioxygenase (locus tags: RPPS3_16850, RPPS3_37890, RPYSC3_16530, and RPYSC3_38130), homogentisate 1,2-dioxygenase (RPPS3_46330 and RPYSC3_46820), and hydroxyquinol 1,2-dioxygenase (RPPS3_21770 and RPYSC3_21800) (Supplementary Tables S5 and S6). Genes involved in ring cleavage pathways of homogentisate and phenylacetate were also identified in PS3 and YSC3, such as the *bed* genes (RPPS3_06600 and 06630–06740; RPYSC3_06760 and 06790–06900) (Supplementary Tables S5 and S6), which are associated with anaerobic benzoate degradation^28^. Additionally, CGA009 has been found to be able to use fatty acids and dicarboxylic acids via a conserved gene cluster associated with the fatty acid beta-oxidation pathway^6,29^. Similarly, we observed that the PS3 and YSC3 strains had a complete gene cluster for the fatty acid beta-oxidation pathway. The *R. palustris* pathways involved in degrading aromatic compounds provide not only extraordinary metabolic versatility but also high utility for bioremediation (e.g., methoxylated aromatics and aromatic amides)^7,29,30^ and bioenergy production (e.g., hydrogen gas)^12^. We noticed no monosaccharide (such as glucose, mannose, xylose, arabinose, or fructose) transporter-related gene in any of the three *R. palustris* genomes. A previous study reported that CGA009 has limited ability to grow on sugars due to the absence of genes encoding glucose transporters, fructose transporters or hexokinases in its genome^6^. However, some studies have reported *R. palustris* strains that could consume glucose or fructose for cell growth^13,31,32^. In our previous study^13^, we found that PS3 and YSC3 could use some monosaccharides, such as fructose and glucose. In addition, there are genes encoding multiple sugar ABC transport systems (RPPS3_01220, RPPS3_01230, RPPS3_033890, RPPS3_35020, RPPS3_35050, RPPS3_35450, RPPS3_43670, RPPS3_45360, RPYSC3_01220, RPYSC3_01230, RPYSC3_35200, RPYSC3_35230, RPYSC3_35650, RPYSC3_44090 and RPYSC3_45830) and TonB-dependent transporters in the *R. palustris* genomes (Supplementary Tables S5 and S6). TonB-dependent transporters have been considered to be involved in dietary polysaccharide processing in bacteria^33^. Therefore, we deduced that the multiple sugar ABC transport systems or TonB-dependent transporters are associated with the uptake of single sugar molecules for *R. palustris*. However, this hypothesis remains to be verified.

### Nitrogen fixation and nitrogen utilization

Biological nitrogen fixation is the process via which nitrogen is converted to ammonia by the nitrogenase complex of microorganisms^34^. All known diazotrophs contain at least one of the three closely related subtypes of nitrogenase-related genes: *nif* (encoding molybdenum nitrogenase), *vnf* (encoding vanadium nitrogenase), and *anf* (encoding iron nitrogenase)^35^. According to previous studies, CGA009 was characterized as a nitrogen-fixing bacterium and harbors the above three gene subtypes^6,22^. On the other hand, only *anf* and *nif* nitrogenase-related genes and no *vnf*-related gene were found in the genomes of PS3 and YSC3. Nevertheless, both strains could fix nitrogen under light-microaerobic conditions^13^. According to genomic analysis, these two strains have a gene cluster encoding the nitrate/nitrite transport pathways (RPPS3_21380-21400 and RPYSC3_21410-21430) (Supplementary Tables S5 and S6); however, no explicit nitrate reductase genes were identified in the two genomes. The PS3 and YSC3 strains have genes encoding proteins associated with denitrification (RPPS3_33410, 41010, 14290–14300 and 20700; RPYSC3_41430, 1438–1439 and 20720) (Supplementary Tables S5 and S6), resembling the denitrification-associated genes in the CGA009 strain. In addition to nitrogen fixation, ferredoxin-nitrite reductase, encoded by *nirA* (RPPS3_37380 and RPYSC3_37610), can directly convert nitrite to ammonium, while ammonium can be taken up from the environment via two ammonium transporters (RPPS3_02860 and 02880; RPYSC3_02920 and 02940) (Supplementary Tables S5 and S6). As shown in Fig. 5, ammonium can be assimilated by glutamine synthetase (RPPS3_10270, 30150 and 41690; RPYSC3_10150, 30130 and 42060) and further converted to glutamate by glutamate synthetase (RPPS3_04850 and 08970; RPYSC3_04930 and 09180) (Supplementary Tables S5 and S6) for amino acid metabolism.

### Root colonization

Root colonization by bacteria is regarded as an essential step for PGPRs to promote plant growth^3^. We compared genes associated with root colonization, such as genes involved in chemotaxis, cell motility, and biofilm formation^36^. All three strains possess three sets of the *che* genes (i.e., *cheA*, *cheB*, *cheR*, *cheW*, and *cheY*) (Supplementary Fig. S2). Cluster I comprises eight chemotaxis genes, which is the most complete cluster. Moreover, both PS3 and YSC3 contain the *che*Z gene (RPPS3_11990 and RPYSC3_11730), which is annotated as a hypothetical protein in CGA009^6^. Genes encoding methyl-accepting chemotaxis proteins (RPPS3_00100, RPPS3_04430, RPPS3_11420, RPPS3_17980, RPPS3_36270, RPPS3_42600, RPPS3_44180, RPYSC3_00110, RPYSC3_18180, RPYSC3_36490, RPYSC3_42970, RPYSC3_44590, TX73_RS00710, TX73_RS02235, TX73_RS18110, TX73_RS21940, TX73_RS21960, TX73_RS21965, TX73_RS22735, TX73_RS23690 and TX73_RS23695) exist in all three strains. For cell motility, there were 50, 46, and 39 flagella-related genes in PS3, YSC3, and CGA009, respectively (Supplementary Tables S5 and S6 and NC_005296.1). Moreover, we noted that the cell migration rates of the three strains varied substantially. Microscopy showed that PS3 had the fastest migration speed, followed by YSC3. By contrast, CGA009 was almost immobile Supplementary Videos S1–S3). Several genes involved in biosynthesis or transportation of polysaccharides were identified in all three strains, for example, *exo* genes, which are responsible for exopolysaccharide (EPS) biosynthesis^22^. The *lptG* and *lptF* genes are responsible for lipopolysaccharide transportation^37^. Genes known to be associated with biofilm formation (as reported in the KEGG database), such as *pel*, *psl*, and *glg*, were not identified in these *R. palustris* strains; however, all three strains showed the ability to form biofilms during cultivation in PNSB broth (Supplementary Fig. S3a), and CGA009 shown higher biofilm formation than did PS3 and YSC3. In addition, biofilm formation was not significantly different between the PS3 and YSC3 strains (Supplementary Fig. S3).

### Deduced plant growth promotion-related genes

The mechanisms for PGP by rhizobacteria include nitrogen fixation, improvement of nutrient availability, and phytohormone production^3,38^. As described above, all three *R. palustris* strains contain the conserved gene cluster that encodes nitrogenase (Supplementary Fig. S4a), which is consistent with the positive phenotyping result obtained for nitrogen fixation. We also found that the *R. palustris* strains harbor genes encoding nitrite reductase and nitric oxide reductase, which convert nitrite to nitric oxide and nitrous oxide. These strains also contain ferredoxin-nitrite reductase, which can directly reduce nitrite to ammonium (Fig. 5). These findings suggest that via enzymatic conversion*, R. palustris* is able to provide plants with available sources of nitrogen.

Phosphate solubilization is also an important mode of action for plant growth^4^. We identified several genes encoding phosphatases, C-P lyases, inositol-phosphate phosphatases, and organic acids in the genomes of all three strains (Supplementary Fig. S4b). Unexpectedly, in our plate assay, these three strains did not present clear zones around the colonies, which indicates that these strains are unable to solubilize phosphate (Supplementary Fig. S5). Some PGPRs can produce phytohormones to stimulate plant growth^39–41^. In our previous study, we conducted a colorimetric assay to demonstrate that both PS3 and YSC3 were able to produce the plant hormone IAA in the presence of tryptophan^13^. As shown in Fig. 5, these two strains possess most of the genes involved in the biosynthetic pathway of IAA in bacteria^42^. However, some genes encoding essential proteins, such as tryptophan-pyruvate aminotransferase (TAA1), tryptophan aminotransferase (TAM1), aromatic amino acid decarboxylase (DDC), tryptophan 2-monooxygenase (MAO), tryptophan side chain oxidase (TSO) and tryptophan monooxygenase (IaaM), were absent in the genomes of these strains. To corroborate their IAA production ability, we employed a more sensitive and accurate method (HPLC) for evaluation (Supplementary methods). As shown in Supplementary Fig. S6, all of these tree strains can produce IAA in the presence of tryptophan. It remains unclear whether the absence of these genes in the *R. palustris* genomes is an artifact of annotation or whether these bacteria have alternative genes for these functions.

ACC deaminase genes were identified in the genomes of PS3 and YSC3 (RPPS3_24510 and RPYSC3_24830, respectively) (Supplementary Tables S5 and S6) but were absent in the genome of CGA009. *R. palustris* can produce 5-aminolevulinic acid (ALA), which is regarded as an effective compound for PGP under abiotic stress^14,43–45^. The genes *hemO* and *hemA*, which are associated with the biosynthesis of ALA, were identified in all three strains. Detailed information about the genes associated with PGP is shown in Supplementary Fig. S4.

### Effect of root exudates of Chinese cabbage on biofilm formation and relative gene expression levels

**Figure 6:**
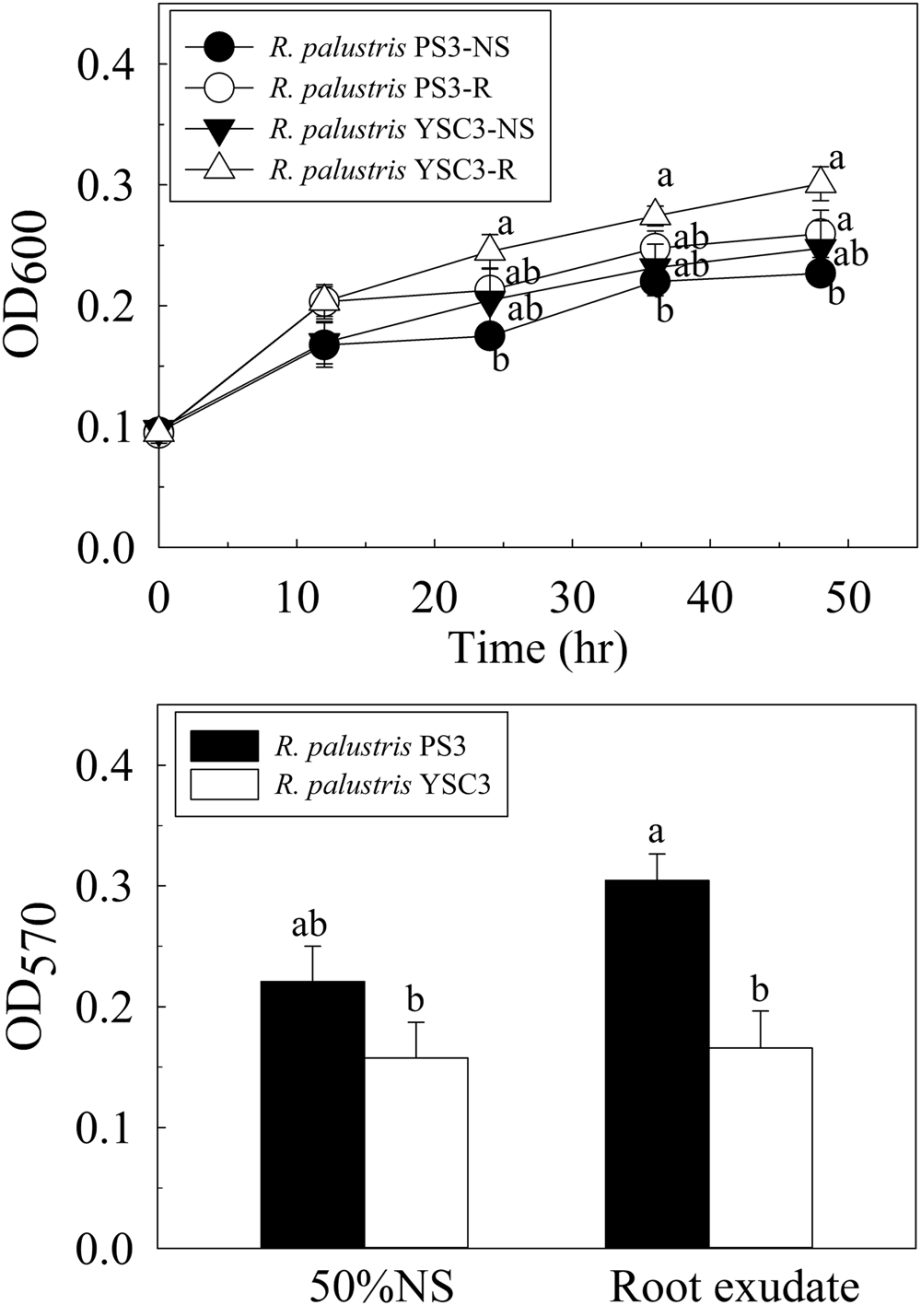
Effects of Chinese cabbage root exudates on growth and biofilm formation of the *R. palustris* PS3 and YSC3 strains. (**a**) Growth curve of *R. palustris* strains in Hoagland NS and root exudate solution. (**b**) Biofilm formation was evaluated by 0.1% crystal violet staining for 15 min at 24 h postincubation. Both *R. palustris* strains were incubated in either half-strength Hoagland NS or root exudate solution (10% (v/v)). The letters indicate statistically significant differences based on Student’s t-test (*P* < 0.05).

Root exudates and their organic acid components are considered important factors for biofilm formation and colonization by PGPRs of the rhizosphere^1^. To elucidate the role of root exudates in microbial activity, we used a hydroponic solution containing root exudates for cultivation of the bacterial strains. As shown in Fig. 6a, the growth rates of both PS3 and YSC3 increased slightly upon treatment with the root exudates (-R) in comparison with those of the control (-NS) groups; however, there was no statistically significant difference at most of the time points. With respect to bacterial biofilm formation, we quantified the crystal violet-stained biofilms at an optical density of 570 nm (OD_570_). As shown in Fig. 6b, there was no significant difference in the accumulation of biofilm between PS3 and YSC3 while they were cultivated in the hydroponic solution. On the other hand, more biofilm was formed by PS3 than by YSC3, while root exudates were supplemented in the hydroponic solution. We also noted that biofilm formation by YSC3 was not altered in the presence of root exudates.

**Figure 7:**
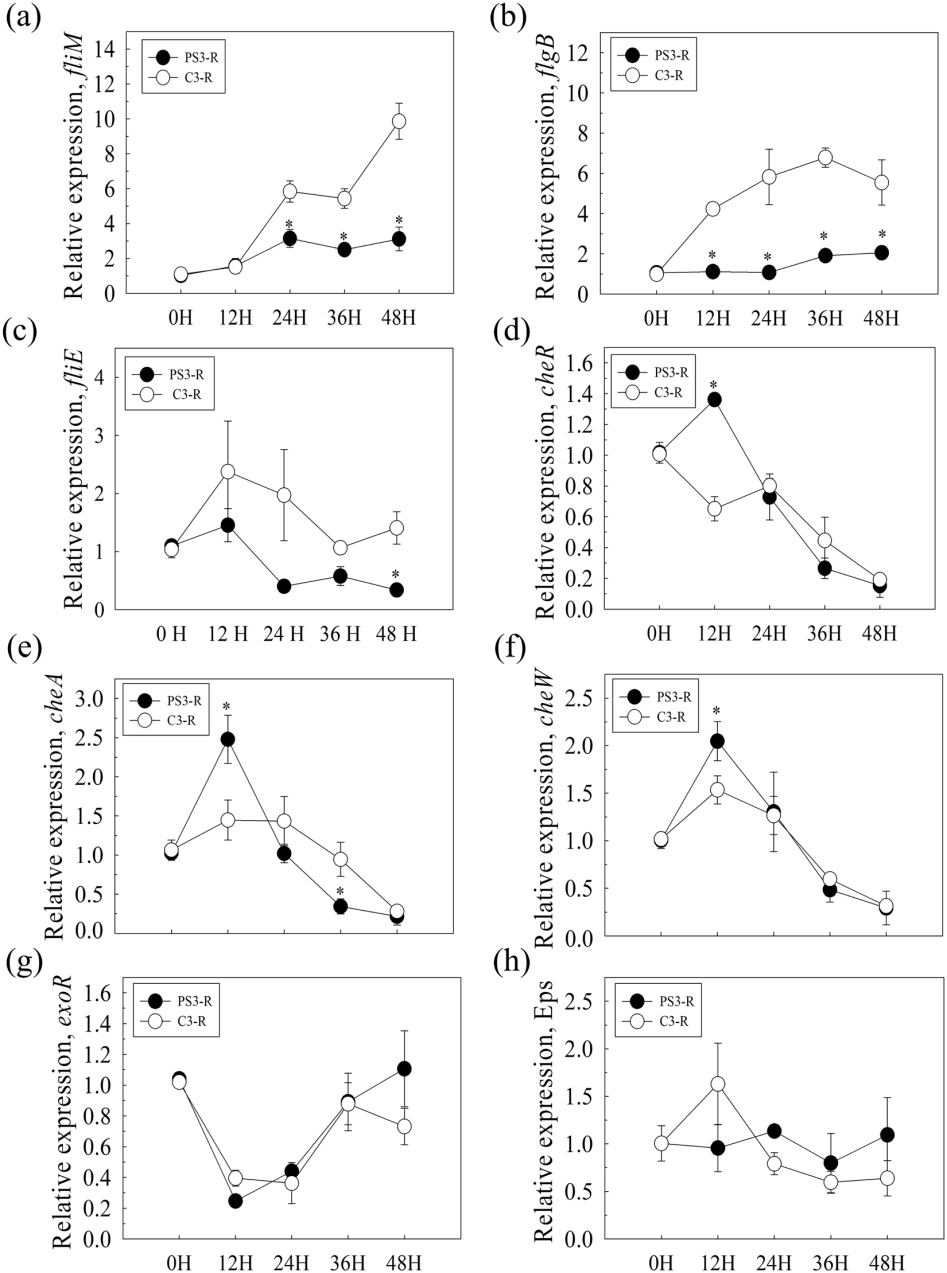
Gene expression patterns of *R. palustris* strains in response to Chinese cabbage root exudate solution. *R. palustris* strains were incubated with Chinese cabbage root exudates, and then, the expression of flagella (*fliM*, *flgB*, *fliE*), chemotaxis (*cheR*, *cheW*, *cheA*) and biofilm formation (Eps and *exo*) genes was determined by qPCR relative to an internal control gene, *clpX*. Relative expression values (SE) were obtained from three biological repeats and measured for three technical repeats. Asterisks indicate significant differences based on Student’s t-test (*P* < 0.05).

The expression patterns of genes associated with bacterial colonization and biofilm formation in response to root exudates were analyzed in a time-course study. As shown in Fig. 7, the relative expression levels of flagella-related genes (Fig. 7a,b) of YSC3 were higher than those of PS3 at most time points during growth. We found that the expression of *fliM* and *flgB* in YSC3 increased with time and that the expression of *fliE* peaked at 12 h and gradually declined. Moreover, according to the cluster integrity, we selected several *che* genes located in cluster I to quantify the expression levels. The results showed that the expression of the chemotaxis-related genes *cheR*, *cheW* and *cheA* of PS3 was upregulated and peaked at 12 h, and these expression levels were significantly higher than those of YSC3 at this time point (Fig. 7d–f). There was no significant difference in the expression of the biofilm formation-related genes *fliE* or *exoR* or the *eps* genes between PS3 and YSC3 (Fig. 7c,g,h).

## Discussion

*R. palustris* strains PS3 and YSC3 were isolated from paddy fields located in neighboring cities in northern Taiwan. These strains have a closer evolutionary relationship than the other characterized *R. palustris* strains (Fig. 2). The chromosomal organization of these two strains is highly conserved, and the strains share 98.59% of their protein-coding genes (Fig. 2c,d and Supplementary Fig. S1 and S7). However, although these strains are closely phylogenetically related, only strain PS3 is able to promote plant growth^13,16^.

While treating individual bacterial cultures with root exudates, substantial differences in biofilm formation and relative gene expression levels were observed between PS3 and YSC3 (Figs 6 and 7). Root exudates contain various carbohydrate-derived compounds (e.g., glucose, maltose, xylose, citric acid, malic acid, and succinic acid)^36,46,47^ and are the most important nutrient sources for rhizospheric microorganisms. In our previous study, we proved that both PS3 and YSC3 can utilize various carbon sources, including the carbon sources mentioned above^13^. Furthermore, many metabolic pathways of the core carbohydrates were identified in the genomes of PS3 and YSC3 (Fig. 5). Therefore, we inferred that these two bacteria are able to utilize carbohydrates derived from root exudates. We noted that the bacterial growth of the PS3 and YSC3 strains was not enhanced in half-strength Hoagland solution containing root exudates (Fig. 6a). We hypothesized that the nutrient levels in this culture medium were too low to sustain normal bacterial growth of *R. palustris*.

In addition to carbohydrates, aromatic hydrocarbons are another type of abundant plant-derived compounds in the rhizosphere. Aromatic hydrocarbons are mainly derived from secondary metabolites (such as flavonoids and phenols) and lignin structures^48^. *R. palustris* can degrade a variety of aromatic compounds^6,7,49^. In the present study, several genes associated with degradation of aromatic compounds were identified in the PS3 and YSC3 genomes. These putative enzymes are involved in either oxygenase-dependent ring cleavage pathways or the anaerobic benzoate degradation pathway (Supplementary Tables S5 and S6). Accordingly, this finding suggests that the *R. palustris* strains can utilize a variety of carbon sources at different levels of oxygen, even under low-oxygen conditions (hypoxia). This result may explain why PS3 can have beneficial effects on plant growth in either soil (aerobic) or hydroponic solution (aerobic-anaerobic interfaces)^13,16^. It would be advantageous for *R. palustris* to act as a PGPR to utilize additional nutrients in agricultural systems under conditions where oxygen demand exceeds supply, such as during flooding of the rice rhizosphere.

Efficient colonization by PGPRs of roots has been considered a key trait conferring beneficial effects to plants, and this ability is closely associated with chemotaxis and biofilm formation^50^. We found that the genetic arrangements of the chemotaxis-related gene cluster (*cheA*, *cheB*, *cheW*, *cheR* and *cheY*) and the flagellar biosynthesis-related gene cluster (*flhA*, *flhB*, *fliP*, *fliQ*, and *fliR*) are very similar among the genomes of PS3, YSC3 and CGA009 (Fig. 5 and Supplementary Tables S5 and S6). Moreover, genes associated with biosynthesis of exopolysaccharides, such as the *exo*, *kps*, and *upp* genes, were identified in the three strains (GenBank: NC_005296, Supplementary Tables S5 and S6). The *upp* gene has already been found to be an important gene in the mediation of biofilm formation by *R. palustris* under photoheterotrophic growth conditions^51^. Although these gene clusters showed very similar genetic arrangements in the three bacteria, the abilities of these strains to form biofilms differed. As shown in Supplementary Fig. S3, CGA009 produced more biofilm than did the other two strains.

Rhizobia are able to supply nitrogen sources to host plants via symbiotic nitrogen fixation in root nodules. In contrast, free-living nitrogen-fixing bacteria release the ammonia synthesized by cells into the environment, and some of this ammonia is converted to nitrite/nitrate^52^. As mentioned, both PS3 and YSC3 have the nitrogenase genes and are able to fix nitrogen under light-microaerobic condition. Upon inoculating these bacteria into the hydroponic nutrient solution (NS) for cultivating plants, we noted that the concentration of ammonia did not change substantially in the NS (data not shown), indicating that free-living N_2_-fixation did not occur under such aerobic culture conditions. Accordingly, we deduced that biological nitrogen fixation is not the primary mode of action for PGP by PS3.

Phosphorus is one of the most important macronutrients, but the availability of phosphorus in soil is limited. Phosphate-solubilizing bacteria can convert insoluble phosphorus (both organic and inorganic) to available forms for plant utilization, and this property is regarded as an essential mode of action for PGP^3,4^. PS3, YSC3 and CGA009 contain many genes involved in phosphate solubilization, such as *phn*, *gcd* and *pqq* (Supplementary Fig. S4b). The *phn* gene family encodes phosphonatases and C-P lyases, which are the enzymes that perform C-P cleavage in organophosphonates^53^. Furthermore, this gene family is also associated with the release of phosphate ions from organic matter, such as fertilizers. On the other hand, genes involved in phosphate solubilization via the production of organic acids were also identified in these *R. palustris* strains. For example, the *gcd* gene, encoding glucose dehydrogenase, and the *pqq* gene, encoding pyrroloquinoline quinone (PQQ), are associated with the production of gluconic acid (GA) in a well-known organic acid-based mechanism of inorganic phosphate solubilization^53^. However, we noted that all three strains lacked orthologs of the *pqqA* gene in the PQQ synthetic pathway (Supplementary Fig. S4b). This finding might suggest that these three strains are unable to synthesize GA. However, it has been reported that *pqqA* is not essential for biosynthesis of PQQ in *Methylobacterium extorquens* AM1^54^. Therefore, the GA production of these three bacteria requires further elucidation. In addition, these bacteria did not harbor related genes encoding phosphatases or phytases, which are the most relevant proteins associated with phosphate solubilization in the environment^53^. Moreover, our data showed that these three strains were not capable of solubilizing inorganic phosphorus from insoluble compounds (Supplementary Fig. S5), even though these strains possess most of the phosphate solubilization-related genes. This result is also consistent with previous reports that *R. palustris* lacks the ability to solubilize phosphate^55,56^. The commonly observed absence of phosphate-solubilization activity among *R. palustris* strains indicates that this function is probably no longer required for sustaining the growth of these bacteria in the environment. Bacteria generally lose some essential biosynthetic functions when the corresponding metabolite is present in sufficient amounts in the bacterial growth environment or is provided by a consortium of organisms^57^. Whether the reduction of metabolic burden for basic cellular processes in *R. palustris* results in adaptive benefits over other genotypes^58^, as well as the causal mechanisms that explain this observation, remains unclear.

We observed that PS3 could modulate root system architecture and promote plant growth^13,16^, indicating the production of phytohormones and other signals during the interactions between *R. palustris* and host plants. It has been demonstrated that enhanced root proliferation, such as increased root size and lateral root number, is closely associated with bacterial IAA levels^39^. IAA biosynthesis in bacteria can be divided into tryptophan-dependent and tryptophan-independent pathways^42^. However, we noted that genes involved in the conversion of tryptophan to other intermediates were absent in the annotation (Fig. 5 and Supplementary Fig. S4c). We identified some genes involved in the synthesis of indole-3-glycerolphosphate and indole in PS3, YSC3 and CGA009; these genes included *trpBA* (RPPS3_00730, RPPS3_00740, RPYSC3_00730, RPYSC3_00740, TX73_RS00360 and TX73_RS00365) and *tnaA* (RPPS3_35930, RPYSC3_36170 and TX73_RS18205). These intermediates are predictive precursors of IAA synthesis in tryptophan-independent pathways^59^, suggesting that these *R. palustris* strains are able to synthesize IAA via a yet-unidentified pathway(s).

Some genes associated with environmental stress tolerance were identified in the genomes of the *R. palustris* strains. Examples of these genes include *acdS* (ACC deaminase; locus tags: RPPS3_24510 and RPYSC3_24830), *hemO*/*hemA* (RPPS3_08610, RPYSC3_08830 and TX73_RS04400 for PS3, YSC3 and CGA009, respectively) and the *hemA* genes (RPPS3_15310, RPYSC3_15390 and TX73_RS04400 for PS3, YSC3 and CGA009, respectively), which encode ALA synthase (Supplementary Fig. S4d). In many PGPRs, such as *Pseudomonas fluorescens*, *Achromobacter piechaudii* and *P. putida*, ACC deaminase can alleviate the detrimental effects of environmental stress and can enhance the stress tolerance of plants by degrading the ethylene precursor ACC^60,61^. ALA is a precursor of porphyrin-containing compounds, such as vitamin B12, chlorophyll, heme and phytochrome^62–65^. Several studies have indicated that exogenous application of ALA can effectively promote plant growth and aid in the stress tolerance of plants^66–69^. For example, Nunkaew *et al*. found that applying the broths of *R. palustris* TK103 and PP803 could promote the growth of rice under high-salt conditions due to the high ALA content^14^.

As described above, PS3 and YSC3 exhibited a very close phylogenetic relationship and shared several conserved regions and genetic arrangements in their chromosomes. The conservation of genetic arrangement is usually used to predict functions of protein-coding genes^70^. Although we identified many putative genes that were associated with known PGP traits in both *R. palustris* strains, only PS3 can successfully promote the growth of plants^13^. Previous studies have indicated that root exudates can regulate transcription in PGPRs^71–73^. As shown in Figs 6 and 7, root exudates of Chinese cabbage had an effect on microbial activities such as biofilm formation as well as on gene expression patterns specific to flagella-related genes (*fliM*, *fliB, fliE*) and chemotaxis-related genes (*cheR, cheW*, and *cheA*) of PS3 and YSC3. The flagella of bacteria are primarily involved in cellular motility but also have sensory functions to sense changes in the environment^74^. Chemotaxis is the movement of bacteria in response to stimuli; bacteria move toward favorable chemicals or away from unfavorable chemicals^75^. Therefore, we deduced that the differences in the effectiveness of PGP by the two bacterial strains were due to the different physiological responses of these strains to specific compounds in the root exudates that act as signal molecules.

Recent studies have indicated that quorum sensing (QS) by PGPRs is involved in biofilm formation, plant colonization and PGP and in triggering induced systemic resistance^76–78^. These beneficial effects are mediated via QS signaling molecules, such as N-acylhomoserine lactone (AHL), which regulate gene expression in response to bacterial population density and interactions with plants^79,80^. Surprisingly, *R. palustris* uses *p*-coumaroyl-homoserine lactone (pC-HSL), an aryl-HSL, as a signaling molecule^81^. pC-HSL synthase was also identified in the genomes of the PS3 and YSC3 strains (RPPS3_03320 and RPYSC3_03390). The synthesis of pC-HSL requires an exogenous source of *p*-coumarate, which is usually present in root exudates^82,83^. Accordingly, we inferred that this aromatic compound triggers the synthesis of the QS molecule of *R. palustris* and mediates interactions with the plant host. pC-HSL is conserved in PGPR strains other than *R. palustris*, such as *Bradyrhizobium* BTAi1^81^. Therefore, it is possible that *R. palustris* can use pC-HSL to have beneficial effects on plant growth.

## Summary

This is the first study to carry out a comparative analysis of *R. palustris* strains that are effective and ineffective in PGP. The PS3 and YSC3 strains are closely related to each other and have similar genomic structures and compositions. Although these strains have many plant growth-promoting genes in common, only the former exhibited PGP. This result suggests that the presence of PGP-associated genes in a bacterium is not sufficient for the bacterium to have beneficial effects on plant growth. Rather, physiological responses to the presence of plant hosts and successful establishment of interactions with the host appear to be critical. To elucidate the underlying molecular mechanisms associated with PGP by *R. palustris* PS3, further experiments are needed. For example, gene deletion and parallel analyses can be used to determine the phenotypic and functional properties associated with plant-bacteria interactions.

## Methods

### Preparation of phototrophic bacterial inoculant

The *R. palustris* strains PS3 and YSC3 are PNSBs and were both isolated from Taiwanese paddy soils^19^. PS3 is an effective PGPR, whereas YSC3 is not. For bacterial inoculant preparation, a single colony was selected and inoculated into 3 mL of PNSB broth as described previously^13^. The culture was then incubated for 24 h at 37 °C (200 rpm). Subsequently, 2.5 mL of these cultures was transferred into 250-mL Erlenmeyer flasks containing 50 mL of fresh PNSB broth. The cultures were incubated under the conditions described above, and the log-phase bacterial cells were harvested for genomic DNA extraction.

### Genomic DNA preparation

A 1-mL suspension of the log-phase bacterial culture was collected in a 2.0-mL Eppendorf tube and centrifuged at 3,000 × g at 4 °C. Subsequently, the supernatant was removed, and the Eppendorf containing the cell pellet was snap-frozen in liquid nitrogen. The cell pellet was homogenized by adding sterile steel beads and rapidly shaking the microcentrifuge tubes back and forth at 9,000 rpm for 1 min in a SH-100 homogenizer (Kurabo, Japan). The homogenization process was repeated three times. The Gentra® Puregene® Kit (QIAGEN) was used for genomic DNA purification according to the manufacturer’s protocol. The quantity and quality of the total DNA were assessed using UV spectrophotometry (Nanodrop ND-1000, J & H Technology Co., Ltd.), and the OD260/280 value of the DNA was higher than 1.80. Agarose gel electrophoresis (0.75%) was used to ensure that the gDNA was intact. Samples containing greater than 25 μg of gDNA were used to perform whole-genome sequencing.

### Whole-genome sequencing

We utilized the MiSeq (Illumina) and the PacBio RSII (Pacific Biosciences) platforms to perform whole-genome shotgun sequencing. The sequencing service was provided by Genomics BioSci & Tech Co., Ltd. (New Taipei City, Taiwan). For Illumina MiSeq sequencing, the DNA library was constructed using the Illumina TruSeq Nano DNA HT Sample Prep Kit according to the TruSeq DNA Sample Preparation protocol (Illumina). This library was diluted and sequenced with 600 paired-end cycles on the Illumina MiSeq instrument by following the standard protocol. For the PS3 strain, the insert size was 500 bp, and 10,471,982 read-pairs and ~3.6 Gb of raw data were obtained; for the YSC3 strain, the insert size was 500 bp, and 11,242,474 read-pairs and ~3.8 Gb of raw data were obtained. For PacBio SMRT sequencing, the DNA library was constructed according to the PacBio SampleNet – Shared Protocol (Pacific Biosciences). After dilution, the library was loaded onto the instrument with the DNA Sequencing Kit 4.0 v2 (part number PB100-612-400) and a SMRT Cell 8 Pac for sequencing. Primary filtering analysis was performed with the RS instrument, and secondary analysis was performed using the SMRT analysis pipeline, version 2.1.0. For the PS3 strain, the average length of the reads was 7,112 bp, and 164,831 reads and ~1.1 Gb of raw data were obtained; for the YSC3 strain, the average length of the reads was 6,342 bp, and 192,795 reads and ~1.2 Gb of raw data were obtained.

### *De novo* genome assembly

The *de novo* genome assembly was based on the paired-end Illumina reads and the PacBio reads. The raw Illumina reads were trimmed at the first position from both the 5′- and 3′-ends that had quality scores lower than 20 by the software Trimmomatic^84^. After discarding these reads, all Illumina reads were shorter than 210 bp, and high-quality sets of 10,470,949 (PS3 strain, ~2.6 Gb of raw data) and 11,241,446 read-pairs (YSC3 strain, ~2.8 Gb of raw data) were obtained. These trimmed reads were individually matched with the corresponding PacBio reads and used as the input for SPAdes Genome Assembler, version 3.5^85^, with default parameters. Finally, the whole-genome sequences were obtained, and the genomic sizes of PS3 and YSC3 were 5,269,926 bp and 5,371,816 bp, respectively.

### Genome annotation

Annotations of the PS3 and YSC3 genomes were based on the procedures described by Cho, *et al*.^86^. The programs RNAmmer^87^, tRNAscan-SE^88^, and PRODIGAL^89^ were used for gene prediction. The genomic sequence of *R. palustris* CGA009^6^ was used as the reference, and the initial annotation of each protein-coding gene was conducted by OrthoMC^90^ with a BLAST^91^ e-value cutoff of 1e-15 and an inflation value of 1.5. Then, BLASTP^91^ searches against the NCBI non-redundant (nr) protein database, BlastKOALA^92^, and the PATRIC platform^93^ were used for manual curation to improve the annotation. For functional categorization, all protein-coding genes were used to run BLASTP^91^ searches against the COG functional category database as described by Galperin, *et al*.^94^ with an e-value cutoff of 1e-10. The program CIRCOS^95^ was used to plot the gene locations, GC-skew and GC content.

### Phylogenetic analysis

To infer the relatedness among the *R. palustris* strains, phylogenetic trees were constructed based on MLSA and *puf* genes. For MLST analysis, three housekeeping genes, *recA*, *rpoB* and *dnaK*, were selected. The sequences of these three genes were retrieved from GenBank. Then, individual gene sequences were validated by alignment using ClustalW multiple alignment program^96^ with the default settings. Subsequently, these genes were combined to form a *recA-rpoB-dnaK* concatenated sequence by BioEdit^97^. MEGA7.0.14^98^ was used to construct the topological tree using the maximum likelihood program^99^. The general time-reversible model and gamma distributed with invariant model (GTR + G + I)^100^ were evaluated from the alignment in the maximum likelihood framework. To estimate the level of support for each branch, the 1,000 bootstrap^101^ samples of the alignment were generated by using the maximum likelihood program^99^ in MEGA7.0.14^98^. The *puf* genes consisted of *pufL* and *pufM*, which encode the core proteins of the photosynthetic reaction center. The sequences of the *puf* genes from other *R. palustris* strains were downloaded from GenBank. The individual gene sequences were aligned using BioEdit^97^ with the ClustalW multiple alignment program^96^ with the default settings. After gene concatenation, the resulting multiple sequence alignment was used to construct the phylogenetic trees by using the maximum likelihood program^99^ with the general time-reversible model and gamma distributed with invariant model (GTR + G + I)^100^. The 1,000 bootstrap^101^ replicates were used to estimate the level of support for each internal branch.

### Comparative genomic analyses

We performed comparative genomic analyses of the genomes of the PS3 and YSC3 strains and the sequenced *R. palustris* type strain CGA009^6^. Pairwise alignments of all three strains were carried out by MUMmer version 3.23^102^ with default parameters. The conserved genes and homologous gene clusters were identified using OrthoMCL^90^ with the same settings as described above. Genes involved in metabolic pathways were analyzed by KEGG Mapper. Genomic island prediction was performed by Zisland Explorer^24^ with default parameters.

### Collection of Chinese cabbage root exudate solution

To collect the root exudates, Chinese cabbage seeds (*Brassica rapa* L. ssp. *chinensis*, “Maruba Santoh”) were selected and purchased from Formosa Farming Materials Co., Ltd. (Taipei, Taiwan). The seeds were immersed in 70% alcohol for 2 min and then in 3% hydrogen peroxide solution for 7 min for surface sterilization. Then, the seeds were washed thoroughly with sterile distilled water and germinated for 1 day at 25 °C in the dark. Subsequently, well-germinated seeds were transferred to soaked cotton and cultivated under continuous (24-h photoperiod) light-emitting diode lighting (~210 μmol m^−2^s^−1^). After one week, the seedlings were transferred to hydroponic tanks (35 L) in a plant factory facility (College of BioResources and Agriculture, National Taiwan University). Twenty-four seedlings were cultivated in each of the tanks, which were equipped with air pumps to homogenize the solution and maintain the dissolved oxygen. Half-strength Hoagland solution was used as an NS (0.255 g L^−1^ KNO_3_, 0.245 g L^−1^ MgSO_4_•7H_2_O, 0.04 g L^−1^ NH_4_NO_3_, 0.034 g L^−1^ KH_2_PO_4,_ 11.25 mg L^−1^ Fe-EDTA, 1.43 mg L^−1^ H_3_BO_,_ 0.0255 mg L^−1^ CuSO_4_, 0.11 mg L^−1^ ZnSO_4_•7H_2_O, 0.95 g L^−1^ MnCl_2_•4H_2_O, 0.06 mg L^−1^ Na_2_MoO_4_•2H_2_O and 0.59 g L^−1^ Ca(NO_3_)_2_•4H_2_O)^103^. The initial pH value was adjusted to 6.0 by H_3_PO_4_. After 7 days of cultivation, the NSs containing root exudates were collected and filtered through 0.22-μm filter membranes. The sterility of these root exudate solutions was checked by plating onto nutrient agar plates.

#### Effect of root exudates on the growth of *R. palustris* strains

A single bacterial colony was selected, inoculated into 3 mL of PNSB broth as described previously^13^, and incubated for 24 h at 37 °C (200 rpm). Then, the absorbance at 600 nm was adjusted to 1.0 using fresh PNSB broth, and 0.5 ml of the above broths were inoculated into 250-mL Erlenmeyer flasks containing 50 mL of fresh 50% Hoagland solutions as well as the root exudate solutions described above. The growth concentration was determined by measuring the optical density at 600 nm (OD_600_) using a spectrophotometer (Ultrospec 2100 pro, Amersham Biosciences).

#### Effect of root exudates on the biofilm production of *R. palustris*

Biofilm formation assay was performed according to a protocol proposed by Tram, *et al*.^104^ with some modifications. A single bacterial colony was selected, inoculated into a 10-mL sterile plastic tube containing 3 mL of PNSB broth^13^, and then incubated at 37 °C and 200 rpm for 24 h. Hoagland solution was used in this experiment as the hydroponic solution. The above culture broth (20 μL) was taken and inoculated into a 96-well plate in which each well contained 180 μL of Hoagland solution with or without root exudates. Bacteria grown in the wells were incubated at 37 °C under stirring (500 rpm) in darkness. For biofilm quantification, the broth in the well was slowly emptied and then rinsed with sterile distilled, deionized water (DDW) to remove the incomplete biofilm. The plate was air dried for 5 min, and the biofilm was then stained with 200 μL of 0.1% crystal violet for 15 min. The crystal violet solution was removed, and the well was washed with DDW three times prior to observation. Subsequently, 200 μL of 95% ethanol was added into the well and shaken vigorously by vortex. Following, the absorbance at 570 nm was determined by using a multilabel reader (VICTOR3 1420-050, PerkinElmer).

#### Gene expression analysis of *R. palustris* in response to root exudates

*R. palustris* broth was inoculated (10% (v/v)) into 200 mL of half-strength Hoagland solution and 200 mL of the abovementioned root exudate solution. The cells were then incubated at 25 °C and 150 rpm in the dark. Bacterial cells were sampled at different time intervals for RNA extraction. Bacterial cell pellets were collected by centrifugation (5000 × g for 5 min at 4 °C). Subsequently, the cell pellets were snap-frozen in liquid nitrogen and homogenized by adding sterile steel beads and rapidly shaking the microcentrifuge tubes back and forth at 9,000 rpm for 1 min in an SH-100 homogenizer (Kurabo, Japan). The homogenization process was repeated three times. RNA purification was performed by the Direct-zol^TM^ RNA MiniPrep Kit (Zymo Research, USA) according to the manufacturer’s instructions. For reverse transcription, 2 μg of total RNA was reacted with random hexamers and the SuperScript III reagent to synthesize first-strand cDNA according to the protocol for SuperScript® III Reverse Transcriptase (Invitrogen, USA). Quantitative PCR analysis of gene expression was performed by using the SYBR Green Real-Time PCR Master Mix Kit (Kapa Biosystems, USA), and the fluorescence intensity was measured by the LightCycler 480 system (Roche, Germany). The real-time PCR conditions were as follows: denaturation at 95 °C for 3 min, followed by 45 cycles of 95 °C for 10 s, 60 °C for 20 s and 72 °C for 1 s. A program of 95 °C for 5 s and 60 °C for 1 min was used to obtain a melting curve. All qPCR analyses were performed in three biological replicates. The housekeeping gene *clpX* was used as the reference gene for transcript normalization. All primer pairs used for quantitative RT-PCR are listed in Supplementary Table S8. The fold change in the expression of target genes in each treatment was calculated using the 2^−ΔΔCt^ method^105^.

### Statistical analysis

Analyses of variance were performed with *R* version 3.4.3. Fisher’s least significant difference (LSD) test was used for multiple range analyses to determine significant differences between groups of data. The results were considered significant at *P* < 0.05.

## Electronic supplementary material

Supplementary materials and methods

Supplementary Video S1

Supplementary Video S2

Supplementary Video S3

Supplementary Table S1

Supplementary Table S2

Supplementary Table S3

Supplementary Table S4

Supplementary Table S5

Supplementary Table S6

Supplementary Table S7

Supplementary Table S8

## Data Availability

The data associated with genomic information for *R. palustris* PS3, YSC3 and CGA009 strains in this paper are available in the NCBI genome database under accession numbers CP019966.1, CP019967.1 and NC_005296.1, respectively.
