# Supplementary material for: Whole-genome sequencing and comparative analysis of two plant-associated strains of Rhodopseudomonas palustris (PS3 and YSC3)
Source: Sci Rep. 2018 Aug 24;8:12769. doi: 10.1038/s41598-018-31128-8 (PMC6109142; doi:10.1038/s41598-018-31128-8)
Supplement: Supplementary file 1 — Supplementary materials and methods [file 41598_2018_31128_MOESM1_ESM.pdf]

Whole-genome sequencing and comparative analysis of two plant-associated strains of *Rhodopseudomonas palustris* (PS3 and YSC3)

Kai-Jiun Lo[1], Shih-Shun Lin[1,2,6,7], Chia-Wei Lu[8], Chih-Horng Kuo[3,4,5*], Chi-Te Liu[1,2*]

[1] Institute of Biotechnology, National Taiwan University, Taipei, 106, Taiwan.
[2] Agricultural Biotechnology Research Center, Academia Sinica, Taipei, 115, Taiwan.
[3] Institute of Plant and Microbial Biology, Academia Sinica, Taipei, 115, Taiwan.
[4] Molecular and Biological Agricultural Sciences Program, Taiwan International Graduate Program, National Chung Hsing University and Academia Sinica. Taipei, 115, Taiwan.
[5] Graduate Institute of Biotechnology, National Chung Hsing University. Taichung City, 402, Taiwan.
[6] Center of Biotechnology, National Taiwan University. Taipei, 106, Taiwan.
[7] National Center for High-Performance Computing, National Applied Research Laboratories. Hsinchu, 300, Taiwan.
[8] Center for Shrimp Disease Control and Genetic Improvement, National Cheng Kung University, Tainan, 701, Taiwan.
* Correspondence: Chi-Te Liu; Email: chiteliu@ntu.edu.tw; Chih-Horng Kuo; Email: chk@gate.sinica.edu.tw (GHK)

Supplementary material

Phylogenetic tree analysis of all conserved single-copy genes in *Rhodopseudomonas palustris.*

For the molecular phylogeny based on all single-copy protein-coding genes conserved within the genus *Rhodopseudomonas*, the procedure was based on that described by Cho, et al.[1]. Briefly, OrthoMCL[2] was used for homologous gene cluster identification; gene clusters that contained exactly one entry from each of the *Rhodopseudomonas* genomes were selected. Multiple protein sequence alignment was processed for each individual gene cluster using MUSCLE version 3.8.31[3] and then concatenated. The maximum likelihood phylogenetic inference was performed using

PHYML version 20120412[4]. The proportion of invariable sites and the gamma distribution parameter were estimated from the dataset, and the number of substitution rate categories was set to four. Bootstrap supports were estimated based on 1,000 replicates.

Biofilm formation assay

Biofilm formation assay was performed according to a protocol proposed by Tram, et al.[5] with some modifications. A single bacterial colony was selected, inoculated into a 10-mL sterile plastic tube containing 3 mL of PNSB broth, and then incubated at 37°C and 200 rpm for 24 h. Subsequently, 0.3 mL of the above broth was inoculated into a 10-mL sterile plastic tube containing 3 mL of fresh PNSB broth. This tube was incubated in a static state at 25°C for 5 days. After that, the broth was slowly emptied, and the residual suspension was carefully removed by pipette. The tube was rinsed with sterile distilled, deionized water (DDW) to remove the incomplete biofilm. The tube was air dried for 5 min, and then the biofilm was stained with 4 mL of 1.0% crystal violet for 15 min. The crystal violet solution was removed, and the tube was washed with DDW three times prior to observation. For biofilm quantification, 5 mL of 95% ethanol was added to the above tube and shaken vigorously by vortex. Following, the absorbance at 570 nm was determined by using a multilabel reader (VICTOR3 1420-050, PerkinElmer).

Phosphate-solubilizing activity assay

Qualitative phosphate-solubilizing activity assay was performed according to the protocol proposed by Nautiyal[6]. Bacterial strains were tested by plate assay containing dissolve phosphorus agar (DPA) medium. DPA medium was modified from National Botanical Research Institute's phosphate growth medium (NBRIP) and National Botanical Research Institute's phosphate growth medium devoid of yeast extract (NBRIY) and contained the following (L^{-1}): 10 g of glucose, 5 g of $Ca_3(PO_4)_2$, 5 g of $MgCl_2 \cdot 6H_2O$, 0.2 g of KCl, 0.1 g of $MgSO_4 \cdot 7H_2O$, 0.1 g of $(NH_4)_2SO_4$, 0.002 g of $MnSO_4$, 0.002 g of $FeSO_4 \cdot 7H_2O$ and 15 g of Bacto agar. A single bacterial colony was selected, inoculated into 3 mL of PNSB broth as described previously[7], and incubated for 24 h at 37°C under stirring (200 rpm) in darkness. Subsequently, 2.5 mL of the above broths were transferred into 250-mL Erlenmeyer flasks containing 50 mL of fresh PNSB broth. The cultures were incubated under the same conditions described above. Then, 20 µL of the bacterial cell suspensions was spotted onto DPA plates, which were then incubated at 37°C for 3 days. A *Burkholderia* sp. isolate was used as a positive strain.

Indole acetic acid production of *R. palustris*

A single bacterial colony was selected, inoculated into 3 mL of PNSB broth as

described previously[7], and incubated for 24 h at 37°C under stirring (200 rpm) in darkness. Following, 2.5 mL of the above broths were inoculated into 250-mL Erlenmeyer flasks containing 50 mL of fresh PNSB broth and incubated under the same conditions described above. After two days, the supernatant of the bacterial broth was filtered through a 0.22-μm filter membrane. IAA purification was performed according to a protocol proposed by Lin, et al.[8] with some modifications. The above filtered solution was mixed with the same volume of cold methanol. Then, the mixture was vortexed and stored at -20°C for 30 min. The supernatant was collected by centrifugation at 14,000 rpm and 4°C for 10 min and then was dried by a SpeedVac concentrator (CVE3110, EYELA) at 40°C and 1,000 rpm. Finally, the dried sample was suspended in 25% methanol with 1% acetic acid and then analyzed by high-performance liquid chromatography (HPLC). The HPLC system (600E Model, Waters-Millipore, Milford, MA, USA) equipped with a UV detector (486E, Waters-Millipore, Milford, MA, USA) was used in this study. The reversed-phase C18 column (Phenomenex Luna C18 column, 250×4.6 mm, 5 μm) with mobile phases A (0.1% acetic acid in water) and B (0.1% acetic acid in methanol) was used. Chromatographic separation was carried out by linear gradient elution with a flow rate of 1 mL/min. The gradient program was as follows: 0-30 min from 30% to 80% B and 30-40 min from 80% to 30% B. The total run time was 40 min, and an injection volume of 20 μL was

used. The column temperature was set at 25°C, and the optical density was measured at a wavelength of 280 nm. Pure IAA (Sigma-Aldrich) was use as a standard.

Supplementary references

1 Cho, S. T. *et al.* Genome analysis of *Pseudomonas fluorescens* PCL1751: A Rhizobacterium that controls root diseases and alleviates salt stress for its plant host. *Plos One* **10**, e0140231; 10.1371/journal.pone.0140231 (2015).

2 Li, L., Stoeckert, C. J., Jr & Roos, D. S. OrthoMCL: Identification of ortholog groups for eukaryotic genomes. *Genome Res* **13**, 2178-2189 (2003).

3 Edgar, R. C. MUSCLE: multiple sequence alignment with high accuracy and high throughput. *Nucleic Acids Res* **32**, 1792-1797, (2004).

4 Guindon, S. & Gascuel, O. A simple, fast, and accurate algorithm to estimate large phylogenies by maximum likelihood. *Syst Biol* **52**, 696-704, doi:10.1080/10635150390235520 (2003).

5 Tram, G., Korolik, V. & Day, C. J. MBDS solvent: An improved method for assessment of biofilms. *Adv Microbiol* **3**, 200-204 (2013).

6 Nautiyal, C. S. An efficient microbiological growth medium for screening phosphate solubilizing microorganisms. *FEMS Microbiology Letters* **170**, 265-270 (1999).

7 Wong, W. T. *et al.* Promoting effects of a single *Rhodopseudomonas palustris*

inoculant on plant growth by Brassica rapa chinensis under low fertilizer input. *Microbes Environ* **29**, 303-313 (2014).

8 Lin, G.-H., Chang, C. Y. & Lin, H.-R. Systematic profiling of indole-3-acetic acid biosynthesis in bacteria using LC–MS/MS. *J Chromatogr B* **988**, 53-58 (2015).

Supplementary Figures

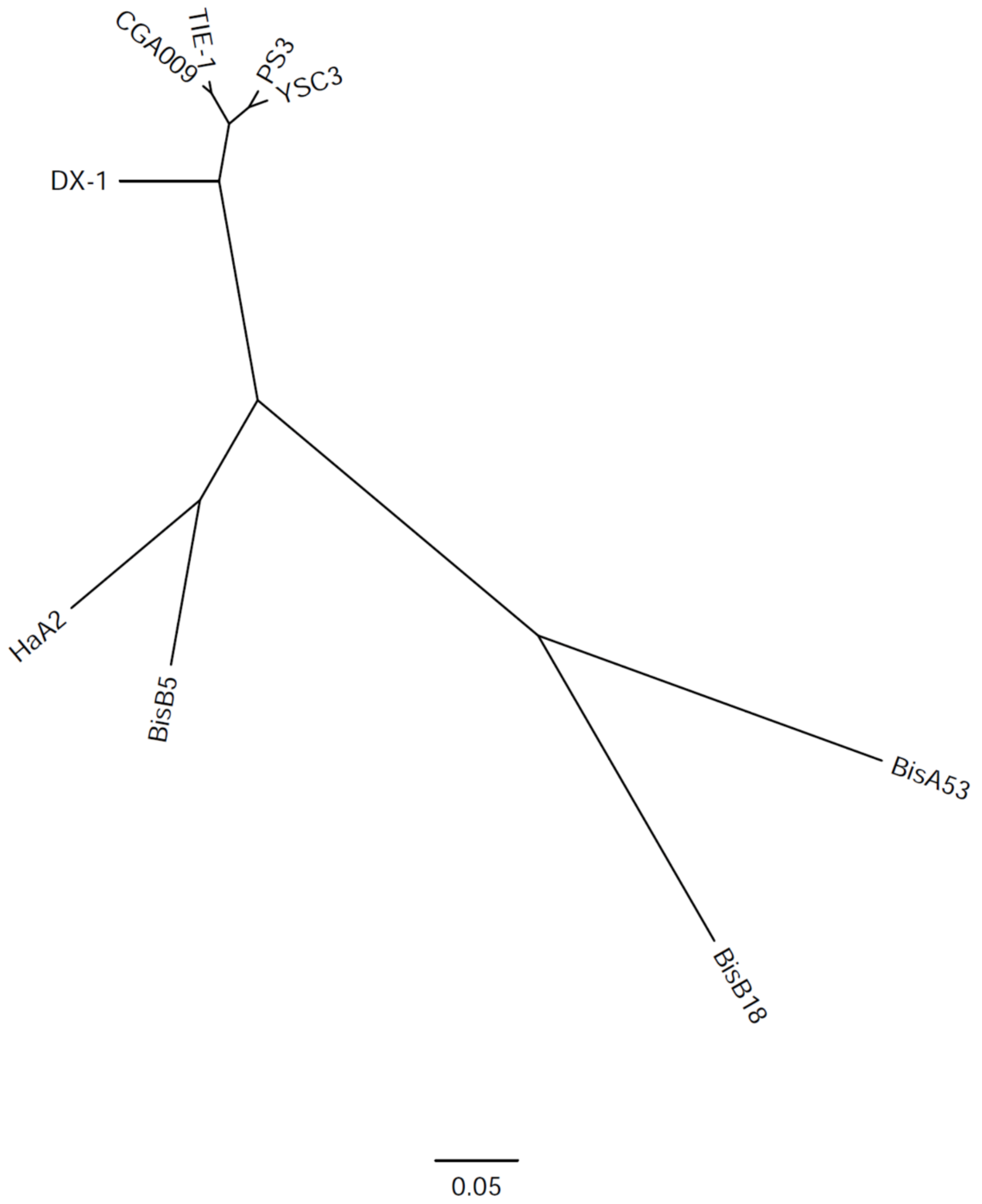

Supplementary Figure S1. Phylogenetic tree based on the concatenated amino acid sequences of 2,515 single-copy conserved genes shared in *R. palustris* strains showing relationships among the genus *Rhodopseudomonas*.

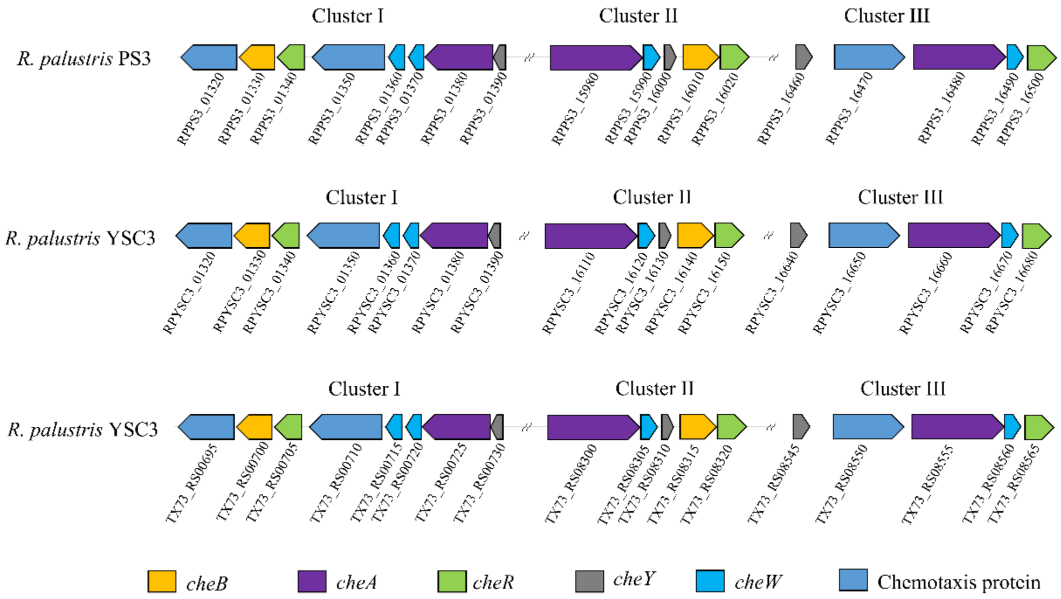

Supplementary Figure S2. Comparison of the chemotaxis (*che*) gene clusters in *R. palustris* PS3, YSC3 and CGA009 strains. The chemotaxis genes were predicted in this study by BLASTP searches against the NCBI non-redundant (nr) protein database and KEGG database.

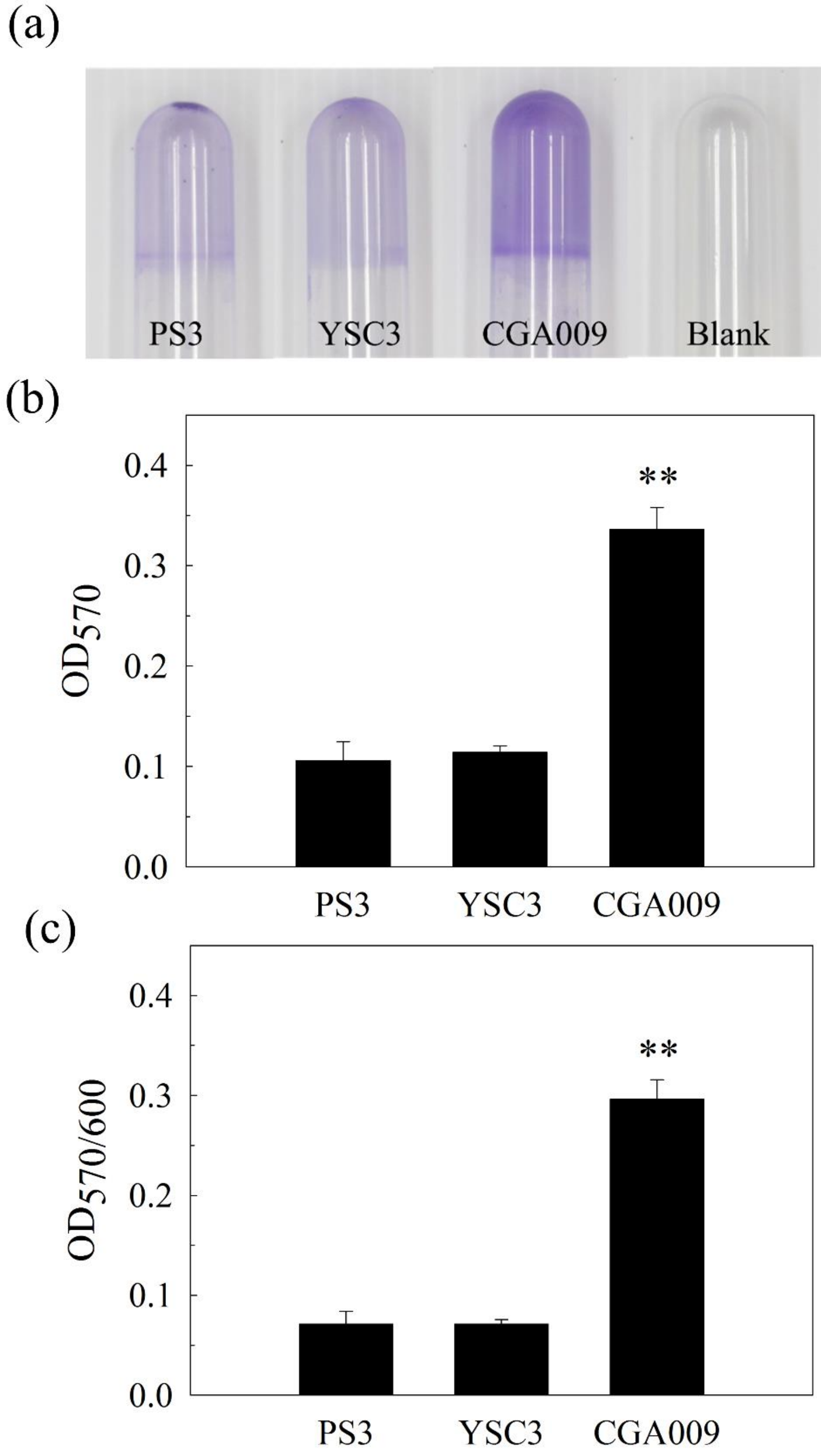

Supplementary Figure S3. Qualification and quantification of biofilm formation in *R. palustris* PS3, YSC3 and CGA009. (a) The photograph of biofilm staining with 1% crystal violet. (b) Biofilm production and (c) biofilm productivity of *R. palustris*

strains.

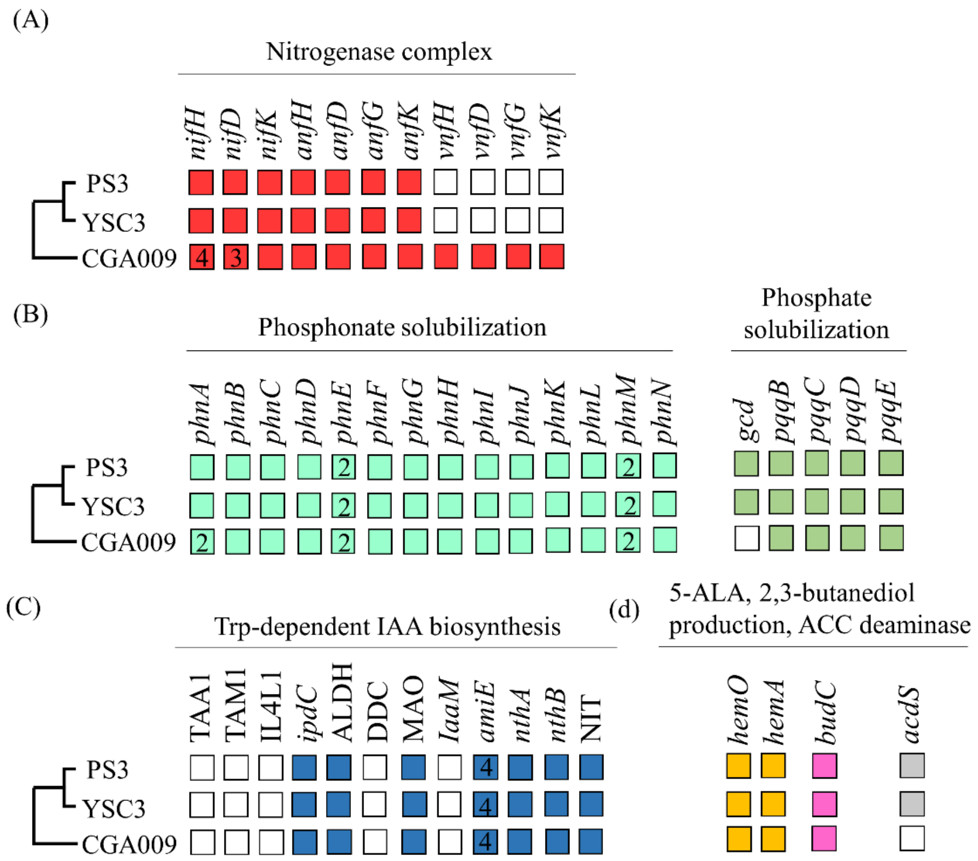

Supplementary Figure S4. Putative genes related to plant growth promotion in the genomes and biofilm production of the PS3, YSC3 and CGA009 strains. A comparative summary of the presence (colored) and absence (empty) of genes for (a) nitrogenases, (b) phosphate solubilization, 5-ALA, 2,3-butanediol production, ACC deaminase and Trp-dependent IAA biosynthesis. The multicopy genes are labeled by their copy number inside the filled square. (c) The biofilm production of *R. palustris* strains in tubes under static condition for 5 days. TAA1, tryptophan-pyruvate aminotransferase; TAM1, tryptophan aminotransferase; IL4L1, amino-acid oxidase; ALDH, aldehyde dehydrogenase; DDC, aromatic amino acid decarboxylase; TSO,

tryptophan side-chain oxidase; MAO, monoamine oxidase; NIT, nitrilase.

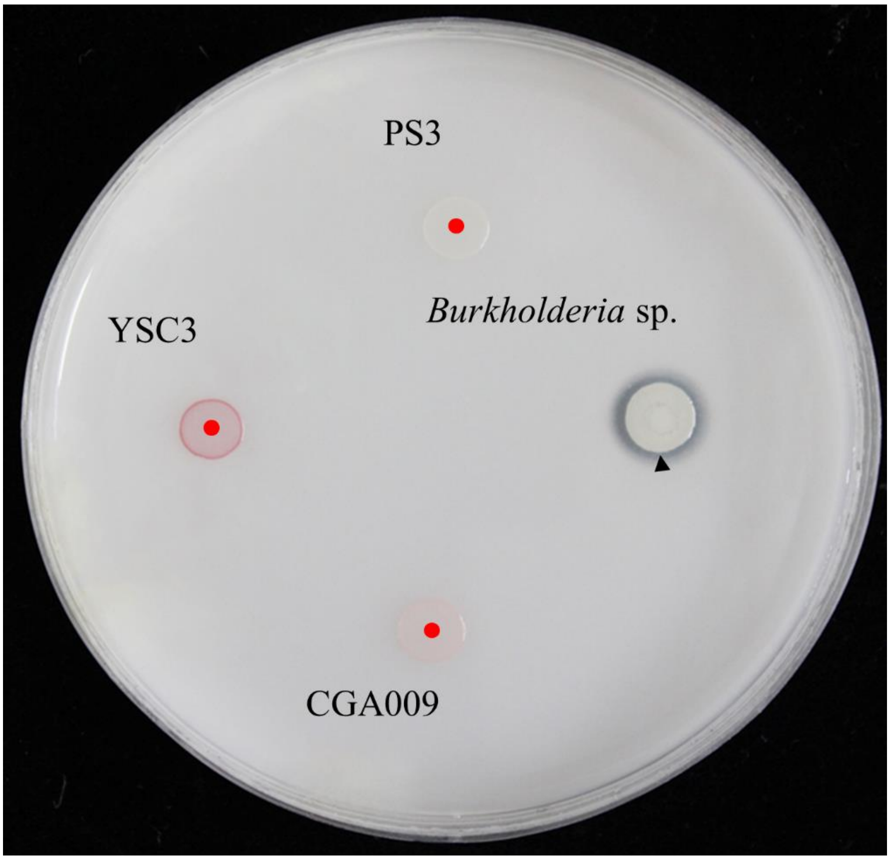

Supplementary Figure S5. The phosphate-solubilizing activity assay of *R. palustris* strains on dissolved phosphorus agar (DPA) medium. The bacterial broths were dropped on the agar surface and then incubated at 28°C for 3 days. *Burkholderia* sp. was used as the positive strain and presented a clear zone around the colony. The red points represent the location of *R. palustris* strains.

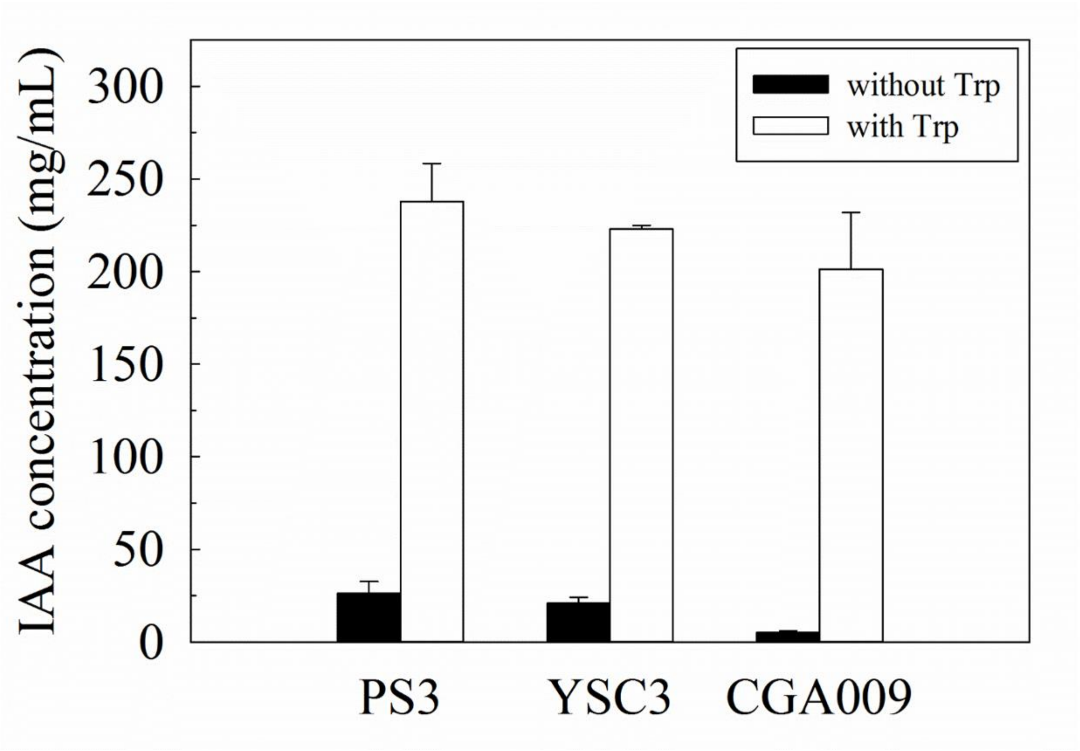

Supplementary Figure S6. Indole acetic acid production of *R. palustris* incubated with/without tryptophan. Indole acetic acid was measured by high-performance liquid chromatography with a C18 reverse column. Bar charts colored in gray and black represent bacteria cultured in PNSB medium with/without tryptophan.

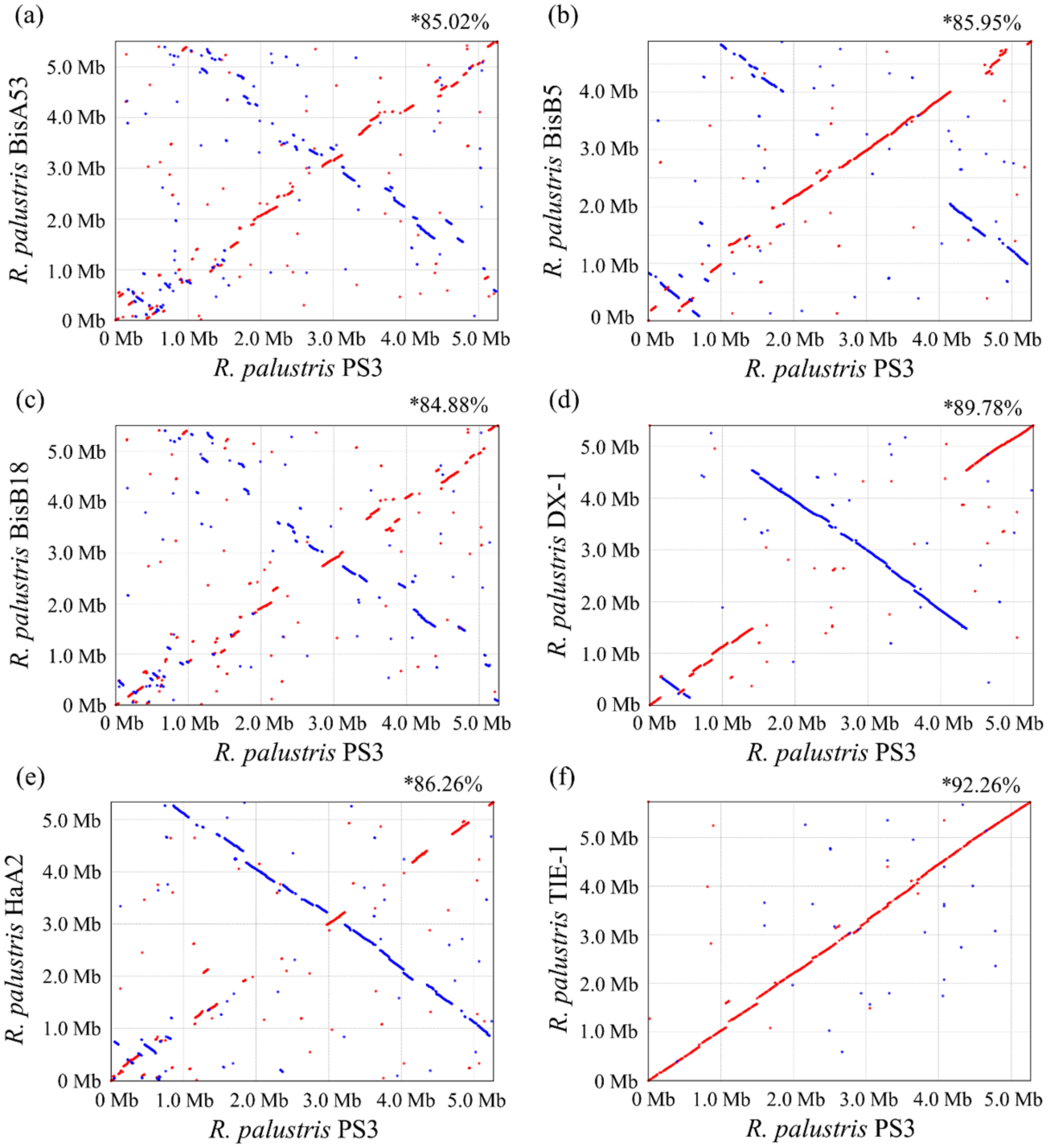

Supplementary Figure S7. Pairwise genome alignments between *R. palustris* PS3 and other related strains. Synteny plots show the comparison of the PS3 genome (each vertical axis) with the genomes of *R. palustris* (each horizontal axis). Forward matches are plotted in red, and reverse matches are plotted in blue. “*” indicates the sequence similarities that were calculated based on nucleotides.

Supplementary video legends

Supplementary Video S1. Cell mobility of *R. palustris* PS3 under a microscope. The bacterial sample was collected from the culture broth that had been incubated for 24 h.

Supplementary Video S2. Cell mobility of *R. palustris* YSC3 under a microscope. The bacterial sample was collected from the culture broth that had been incubated for 24 h.

Supplementary Video S3. Cell mobility of *R. palustris* CGA009 under a microscope. The bacterial sample was collected from the culture broth that had been incubated for 24 h.

Supplementary Table legends

Supplementary Table S1. List of overlapping genes in the *R. palustris* strains PS3, YSC3 and CGA009.

Supplementary Table S2. List of predicted strain-specific genes in the *R. palustris* strain PS3.

Supplementary Table S3. List of predicted strain-specific genes in the *R. palustris* strain YSC3.

Supplementary Table S4. List of strain-specific genes in the *R. palustris* strain CGA009.

Supplementary Table S5. List of predicted genes in the *R. palustris* strain PS3.

Supplementary Table S6. List of predicted genes in the *R. palustris* strain YSC3.

Supplementary Table S7. List of predicted transcriptional regulator in the *R. palustris* strain PS3, YSC3 and GCA009.

Supplementary Table S8. Primers used in this study.
